# Cold and low irradiation shape *Polylepis reticulata*’s seasonal growth and water use dynamics at the Ecuadorian Andean tree line

**DOI:** 10.3389/fpls.2025.1675655

**Published:** 2025-10-13

**Authors:** Aldemar Carabajo-Hidalgo, Daniel Nadal-Sala, Byron Poma, Heidi Asbjornsen, Patricio Crespo, Santiago Sabaté

**Affiliations:** ^1^ Departamento de Recursos Hídricos y Ciencias Ambientales, Facultad de Ingeniería, Universidad de Cuenca, Cuenca, Ecuador; ^2^ Departamento de Biología Evolutiva, Ecología y Ciencias Ambientales, Universitat de Barcelona, Barcelona, Spain; ^3^ CREAF (Centre de Recerca i Aplicacions Forestals), Campus de Bellaterra Universitat Autònoma de Barcelona (UAB) Edifici C., Cerdanyola del Vallès, Spain; ^4^ Department of Natural Resources and the Environment and Earth Systems Research Center, University of New Hampshire, Durham, NH, United States

**Keywords:** high-altitude forests, plant functional traits, *Polylepis reticulata*, sink limitation, tree growth, water use efficiency in growth

## Abstract

**Introduction:**

*Polylepis reticulata* is a tree species that grows in the Ecuadorian Andean páramo at 4500 m a.s.l., an environment characterized by low temperatures, elevated cloudiness, and recurrent fog. These environmental stressors result in scarce periods when both photosynthesis and stem growth can occur. Particularly interesting are *P. reticulata* transpiration, stem growth, and water use efficiency in growth (WUEBAI) dynamics, which may provide valuable insights into the carbon source-sink growth limitation conundrum. Additionally, little is known about *P. reticulata*’s sensitivity to the different environmental drivers, and its related life traits.

**Methods:**

In this study we quantify the seasonal transpiration, stem growth and WUEBAI patterns of *P. reticulata* from continuous field measurements on sap flow and stem growth during an entire year.

**Results:**

We obtained mean annual values of 1.97 ± 0.05 mm2 day-1 for stem growth, 16.47 ± 0.10 dm3 day-1 for transpiration and 1.20 ± 0.05 cm2 m-3 for WUEBAI. Nevertheless, we found a seasonal pattern for these variables. Cold and cloudy conditions led to a reduction in stem growth, with mean values of 1.67 ± 0.12 mm2 day-1 during this season. Concomitantly, transpiration also declined, with mean values of 12.36 ± 0.08 dm3 day-1, reducing WUEBAI, with mean values of 1.35 ± 0.13 cm2 m-3. On the contrary, during the warmer season, when photosynthesis and cambium cell differentiation occurred simultaneously, all the values were higher, with mean stem growth values of 2.58 ± 0.06 mm2 day-1, mean transpiration values of 18.55 ± 0.12 dm3 day-1 and mean WUEBAI values of 1.39 ± 0.16 cm2 m-3, suggesting a sink-driven limitation of tree growth during the cold season.

**Discussion:**

Hence, our results suggest that *P. reticulata*’s growth and transpiration patterns are limited by energy availability, rather than water availability. So, expected temperature increases for the next years may enhance *P. reticulata*’s growth, should water stress not increase.

## 1 Introduction

*Polylepis* is the tree genus that grows at the highest altitude world-wide (Hoch and Körner, 2005). It grows along the Andes mountains of South America from Venezuela to northern Argentina (Kessler and Schmidt-Lebuhn, 2006), with the species *Polylepis reticulata* growing under the extreme climatic conditions of the Ecuadorian páramo, an ecosystem characterized by large daily temperature variations, as well as elevated cloudiness and recurrent fog, that lead to brief periods of high photosynthetically active radiation (PAR). This makes the páramo an energy-limited ecosystem (Carabajo-Hidalgo et al., 2023). Moreover, temperatures are usually low, inhibiting enzymatic reactions (García-Plazaola et al., 2015) and limiting tree growth (Alvites et al., 2019). In response to such energy-limited conditions, *P. reticulata* shows patchy distribution in topographically protected areas with suitable microclimatic conditions, such as high solar radiation and low wind exposure (Toivonen et al., 2017). Conversely, water availability has been found not to limit *P. reticulata*’s transpiration or photosynthesis (Ochoa-Sánchez et al., 2019; Carabajo-Hidalgo et al., 2023), since soil moisture is close to saturation most of the year and annual evaporative demand is well below precipitation (Carrillo-Rojas et al., 2019).

Forests in highland mountain areas, such as the Ecuadorian páramo ecosystem, are among the most sensitive to climate variations (Buytaert et al., 2011; Diaz et al., 2014). Therefore, understanding the ecophysiology of *P. reticulata* can provide valuable information to anticipate responses of the Ecuadorian páramo ecosystem to warming and drought (Kimball and Weihrauch, 2000). To better understand the mechanisms allowing *P. reticulata* to thrive in the Ecuadorian páramo and help to anticipate its potential responses to climate change, it is important to determine the environmental drivers that control its growth and water use, especially the conditions under which transpiration translates into stem-growth. Furthermore, given the paramo’s extreme growing conditions, understanding *P. reticulata*’s growth and gas exchange dynamics can provide valuable insight about the ongoing debate about whether tree growth is primarily driven by photosynthetic uptake (carbon source) or cambial cell inactivation (carbon sink) limitations (Körner, 2015; Cabon et al., 2022).

Water use efficiency in growth (WUE_BAI_) can be explored by studying ratio of transpiration to basal area increment (BAI) (Nadal-Sala et al., 2017), to address the environmental drivers promoting wood formation, and under which circumstances such wood formation is most costly in terms of transpiration. The value of using WUE_BAI_ to explore these relationships, rather than leaf-level gas exchange water use efficiency (iWUE), is that the former refers to both carbon uptake and carbon allocation dynamics, which can be related to growth limitations other than photosynthetic uptake limitation (e.g. cambium inactivation due to low water potentials or low temperatures), whereas the latter refers only to gas exchange at the leaf level, that is, gross productivity (Peñuelas et al., 2011; Wang et al., 2012; Kannenberg et al., 2020). Under favorable circumstances, when carbon uptake and growth are coupled, one would expect coupled WUE_BAI_ and iWUE patterns. Contrastingly, at decreasing temperatures, when cambium is inactivated but photosynthesis is still active, they are expected to decouple.

On the other hand, studies of plant structural and functional traits could give relevant information about the selection pressures experienced in the harsh conditions of Ecuadorian páramo ecosystem, since plant life traits determine how plants respond to environmental factors (Kattge et al., 2019). It is known that variation in traits with climate provides an indicator of plant resource use (Cox et al., 2024), e.g., specific leaf area (SLA) is in many cases positively related with growth rate (Cornelissen et al., 2003), foliar N content is related with photosynthetic potential, and δ^13^C is related to iWUE (Cox et al., 2024). In general, in warm climates and low altitudes, trees are tall, with large and thin leaves, whereas in cold climates and high altitudes, trees are shorter, with smaller and thicker leaves (Hertel and Wesche, 2008; Segovia-Salcedo et al., 2018). In agreement, Macek et al. (2009) found that *P. rugulosa* leaves were larger at lower altitudes, but the opposite was found for other *Polylepis* species, suggesting that not only temperature limitation, but also water limitation could determine plant life traits. Other functional strategies have been reported for *Polylepis* trees, such as photoprotective mechanisms to cope with high ultraviolet radiation or photosynthetic apparatus highly specialized to adapt to low temperatures (González et al., 2007; García-Plazaola et al., 2015). *Polylepis reticulata* is able to grow despite the low energy availability in Ecuadorian páramo ecosystem, as reflected in its highly specialized photosynthetic apparatus that can take advantage of short, intense high-radiation periods to fulfill its metabolic carbon requirements (Carabajo-Hidalgo et al., under review).

The aim of this study was to evaluate seasonal WUE_BAI_ dynamics and iWUE of *P. reticulata*. To do so, we combined field measurements of sap flow (Carabajo-Hidalgo et al., 2023) and stem radial growth to address seasonal WUE_BAI_ dynamics in a *P. reticulata* forest located in the Southern Ecuadorian Andes. Additionally, we conducted a field monitoring campaign to characterize several key plant life traits, namely tree and leaf morphological traits, leaf nutrient content, and whole leaf carbon isotope composition to determine iWUE.

Our specific objectives were: (1) to determine *P. reticulata*’s seasonal stem growth and water use efficiency in growth, to (2) link these variables to the environmental conditions driving such seasonality, and finally (3) to characterize the key functional traits related to water use in *P. reticulata* trees. We hypothesized that i) *P. reticulata* will present a clear seasonality in its stem growth related to temperature limitations, despite the lack of clear growth rings; ii) *P. reticulata* WUE_BAI_ will also exhibit a strong seasonality in water use efficiency in growth, with higher values occurring during warmer periods, when elevated photosynthesis can translate into stem growth; and iii) adaptation to recurrent low temperatures have likely shaped *P. reticulata*’s life traits, and we will find low tree height and high leaf thickness, contrasted with low δ^13^C values and high SLA due to the water availability in the site.

## 2 Materials and methods

### 2.1 Experimental site

The study was conducted within a *P. reticulata* forest patch at 3800m a.s.l., encompassing an area of 15633 m^2^, in the Zhurucay Ecohydrological Observatory (3°04’S, 79°14’W), located in the Pacific side of the Andean cordillera in southern Ecuador. The Zhurucay micro-catchment has a drainage area of 7.53 km^2^ and its land cover is typical of Ecuadorian páramo grasslands, characterized by tussock grasses (mainly *Calamagrostis intermedia*), cushion plants (mainly *Plantago rigida*), planted pine trees (*Pinus patula*), pastures, and patches of *Polylepis* forest, which represent 2% of the total land cover (Mosquera et al., 2015; Correa et al., 2016). The dominant soils in the micro-catchment are Andosols (72% of the area), Histosols (24%) and Leptosols (4%), with *Polylepis* trees commonly covering the organic-rich Histosols (Correa et al., 2017).

Regarding microclimatic conditions, mean annual precipitation was 1298mm, mean annual air temperature was 5.9°C, mean annual relative humidity was 88%, mean annual photosynthetic active radiation was 274.5 µmol m^-2^ s^-1^ and mean annual wind velocity was 3.55m s^-1^, considering a 2-year period measurement from 2018-2019 (Carabajo-Hidalgo et al., 2023).

### 2.2 Whole tree (E_tree_) transpiration data

In order to obtain the whole-tree transpiration we measured tree sap flow following the Heat Ratio Method (HRM) (Burgess et al., 2001) as described in Carabajo-Hidalgo et al. (2023). In short, we installed four self-made sensors with two temperature probes placed equidistant above and below a heater in *P. reticulata* trees and recorded the measurements every 15min in a data logger (CR1000, Campbell Scientific, Inc.) from February 1^st^ to December 31^st^ 2019. Zero flows were determined from the ratio of the temperature values close to 1 since the equidistant placement of temperature probes around the heater allows for identifying periods of time when sap flow is zero (Lopez et al., 2021). This provided us with sap flow velocity (cm h^−1^). Then, we determined active sapwood area per tree at the end of the experiment by collecting four wood cores, one for each monitored tree, and analyzing sapwood thickness based on difference in wood color and light transmission through the vessels (Vertessy et al., 1995; Hernandez-Santana et al., 2011). Combining sap flow and active sapwood measurements, we obtained tree transpiration (E_tree_, in l h^-1^ tree^-1^) as the product of sap velocity by the cross-sectional area of conducting sapwood for all four trees monitored.

### 2.3 Stem basal area increment (BAI_tree_) and growth-based water use efficiency (WUE_BAI_)

To measure stem radial changes during the February-December 2019 period, we installed four high-resolution automatic point dendrometers (ZN11-T-WP, Natkon, Oetwil am See, Switzerland) to the same trees at which sap flow was being measured. Data were recorded at 15min intervals in a datalogger (CR1000, Campbell Scientific, Inc. Logan, UT, USA) from February to December 2019. The ZN11 T-WP point dendrometers have a resolution of less than 1 µm (Zweifel et al., 2021) and are based on a Linear Variable Displacement Transducers (LVDT sensors) that measure the linear displacement of a sensing rod pressed against the stem bark. This displacement, caused by the radius contraction or expansion of the stem, is converted into an electrical signal (Rossi et al., 2006). We installed the dendrometers at breast height, selecting four healthy *P. reticulata* trees located at the edge of the forest, with stems showing a more evident cylindrical shape and equipped with sap flow sensors, to obtain simultaneous measurements of stem diameter and sap flow. Prior to dendrometer installation, we removed the bark, taking care of not damaging the cambium, to minimize the influence of hygroscopic contraction and expansion (Raffelsbauer et al., 2019). Initial diameter at breast height (DBH) values for the four trees are detailed in **Supplementary Table S1** available as **Supplementary Material**. At the end of the growing season, we calculated accumulated BAI for a period with active growth and for a period with basal growth, avoiding the short-term basal area increments that could be due to changes in stem water. Growth-based water use efficiency (WUE_BAI_, cm^2^ m^-3^) was obtained by dividing tree BAI values for a given period “t” (BAI_tree_, cm^2^ t^-1^) by tree transpiration values during the same period (E_tree_, m^3^ t^-1^), following Equation 1 (Nadal-Sala et al., 2017).


(1) $$WUE_{BAI}=BAI_{tree}\;E_{tree}^{-1}\;.$$

### 2.4 Meteorological and soil water content data

We used meteorological variables and soil water content data measured from February to December 2019. Precipitation (mm) was obtained from the main meteorological station located at ZEO, property of the Department of Water Resources and Environmental Sciences at University of Cuenca, using a precipitation gauge (TE525 Texas Electronics Inc., Dallas, TX, USA) connected to a data logger (CR1000, Campbell Scientific, Inc., Logan, UT, USA) that recorded data at 5-min intervals.

We also obtained data from a micrometeorological station located outside the forest, equipped with an anemometer to measure wind speed at 2.5m above the ground surface (Ws, m s^-1^; Met-One 034BCampbell Scientific, Inc.) and a quantum sensor to measure photosynthetically active radiation, also at 2.5 above the ground (PAR, μmol m^-2^ s^-1^; LI-190, LI-COR Bioscience, Lincoln, NE, USA). Air temperature (temperature,°C) and relative humidity (RH, %) were measured with three Vaisala HMP45 sensors (Vaisala, Woburn, MA, USA), two installed in the edge of the forest and one installed in the interior. Soil volumetric water content (VWC, cm^3^ cm^-3^) was measured with CS616 water content reflectometers (Campbell Scientific, Inc.) installed at five locations in the forest at three different depths: organic superficial horizon (10cm depth), organic medium horizon, where most roots are found (25cm depth) and mineral horizon, over 35cm depth. VPD (kPa) was calculated from air temperature and relative humidity. All environmental variables (except precipitation) and VWC were recorded at 15-min intervals on a data logger (CR1000, Campbell Scientific, Inc.). Mean average values for the main environmental variables during the study period can be found in **Supplementary Figure S1** in **Supplementary Material**.

### 2.5 Carbon isotope analysis and iWUE

The carbon isotope composition of whole leaves were determined using a Flash IRMS elemental analyzer (EA-IRMS, ThermoFisher Scientific Inc., MA, USA) connected to an isotope ratio mass spectrometer (DeltaV-Advantatge, ThermoFisher Scientific Inc., MA, USA). The equipment calculate δ^13^C through comparisons with referent standard gases. For the calculation of the iWUE we followed Bauters et al. (2020). First, we used the classic model of C isotope discrimination during photosynthesis to derive leaf Δ^13^C from δ^13^C_a_ and the leaf δ^13^C, which were obtained via the bulk leaf δ^13^C measurements in Equation 2 (Farquhar et al., 1982; Bauters et al., 2020):


(2) $$\Delta 13C_{leaf}=\frac{\delta^{13}C_{a}\;-\;\delta^{13}C_{leaf}}{1+\delta^{13}C_{leaf}}\;.$$

The CO_2_ concentration in the stomatal cavity (Ci) was calculated as follows in Equation 3:


(3) $$C_{i}=\frac{C_{a}*\;{(}^{13}C_{leaf}\;-\;a)}{b\;-\;a}\;.$$

Where C_a_ is the atmospheric value *in-situ* (C_a_ = 411.42 μmols m^-2^ s^-1^), the *a* term is the fractionation during CO_2_ diffusion through the stomata (*a* = 4.4‰ (O’Leary, 1981; Bauters et al., 2020)) and the *b* term is the fractionation associated with reactions by Rubisco and phosphoenolpyruvate carboxylase (*b* = 27‰ (Farquhar and Richards, 1984; Bauters et al., 2020)). Finally, we obtained iWUE with Equation 4, as WUE is related to the ratio of photosynthesis (A) to stomatal conductance (g_s_).


(4) $$iWUE=\frac{A}{g_{s}}=\frac{C_{a}}{1.6}*(1-\frac{C_{i}}{C_{a}}\;).$$

### 2.6 Plant life traits

We measured tree and leaf traits to explore patterns in plant growth, transpiration and water use efficiency. Tree height was obtained from a clinometer (Suunto PM-5/360 PC Clinometer, Suunto, Vantaa, Finland). Wood density (WD, g cm^-3^) was obtained from water volume displace measurements of tree cores. Leaf thickness (mm) was measured using a manual micrometer in the field. Leaf area (LA, mm^2^) was obtained by scanning and measuring leaves with ImageJ software (ImageJ bundled with 64-bit Java 8.). Specific leaf area (SLA, mm^2^ mg^-1^) was calculated following Equation 5 (Macek et al., 2009).


(5) $$SLA=\frac{LA}{DW}$$

Where DW (mg) is leaf dry weight (dried out at 70°C to constant weight). Leaf chlorophyll content (Chlorophyll, SPAD value transformed to mmol m^-2^) was measured with a chlorophyll meter (Chlorophyll Meter SPAD-502Plus, Konica Minolta Holdings Inc, NJ, USA) with an accuracy of ± 1.0 SPAD units. SPAD units were converted to mmol m^-2^ following Equation 6 from Poorter and Bongers (2006):


(6) Chlorophyll=−112.9 +13.9 * SPAD

Leaf nitrogen and carbon concentrations (N_leaf_ and C_leaf_, mg g^-1^) were determined using an elemental analyzer (EA-IRMS, ThermoFisher Scientific Inc., MA, USA). The same analyzers were used for soil nutrient analyses. Finally, predawn water potential and midday water potential (WP_PD_ and WP_MD,_ -MPa), were measured using a Scholander pressure chamber (Model 600D, PMS Instrument Company, OR, USA).

### 2.7 Data analysis

We investigated the covariation between pairs of plant traits through ANCOVA (ANalysis of COVAriance) analysis. When variables did not meet homoskedasticity and normality requirements, log-transformations were implemented. All data analyses were done in R (R Core Team, 2024), version 4.2.2.

#### 2.7.1 Stem growth daily signal integration

**Figure 1:**
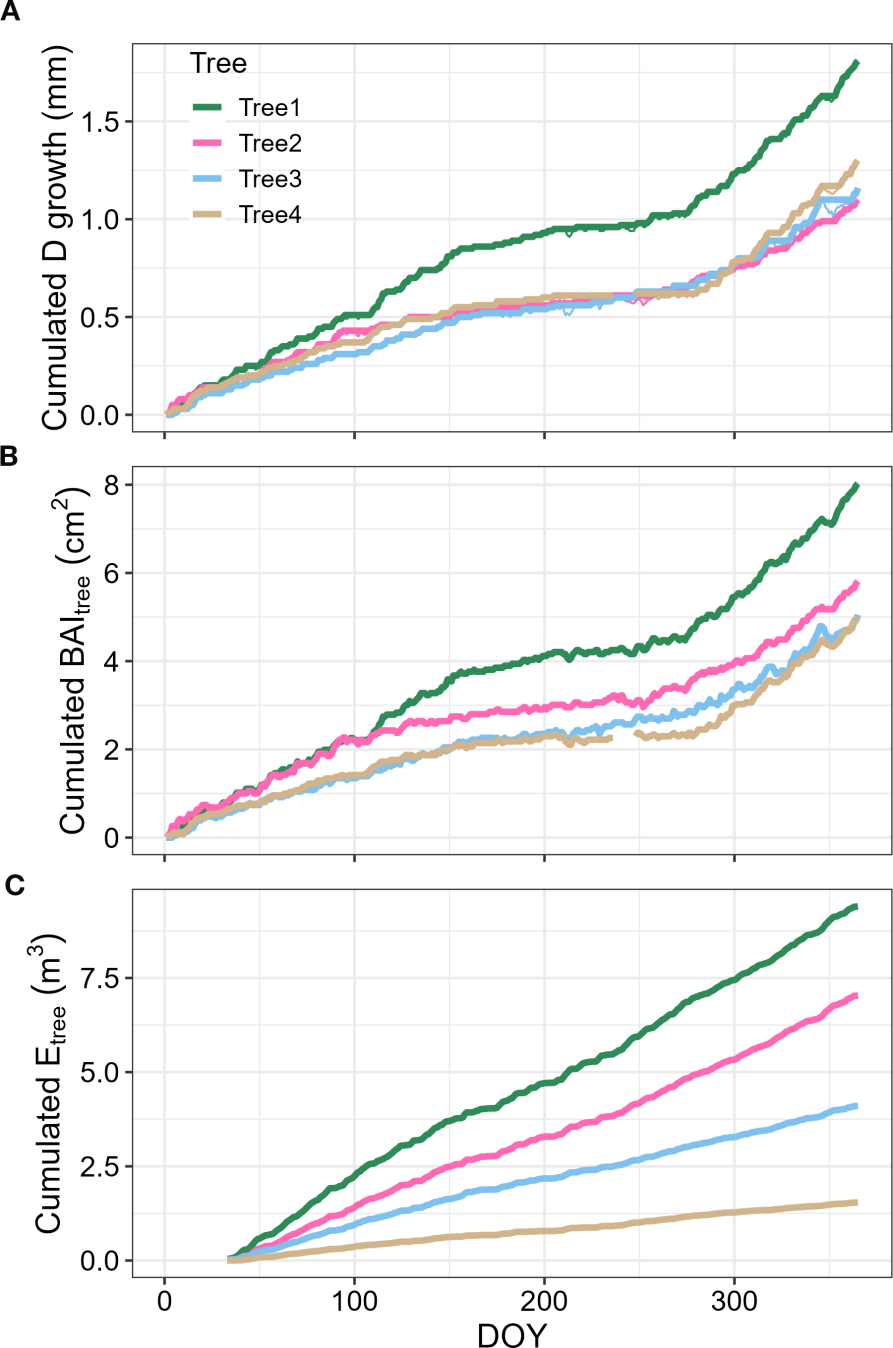
Tree growth and transpiration dynamics for *P. reticulata* growth at páramo (Ecuador) during the year 2019. Panels depict: **(A)** the daily cumulated diameter (D) growth per tree (mm); **(B)** the cumulated daily basal area increment per tree (BAI_tree_, mm^2^); and **(C)** the cumulated daily transpiration (E_tree_, m^3^). Colors depict the values for the four different trees measured.

Daily stem radius change was obtained daily from the 15-minutes stem diameter measurements, recording both the maximum daily value and the minimum daily value. Then, in a similar manner as in Knüsel et al. (2021), we obtained the daily net increase in tree diameter (DTDI, mm) if the maximum radius (MR, mm) for a given day was above any previous daily MR recording, by subtracting the MR to this maximum value. By integrating the DTDI annually, we obtained the cumulated diametric tree growth (mm) and the cumulated annual basal area increment (mm^-2^) (**Figure 1**).

#### 2.7.2 Temporal integration of the growth, meteorological and transpiration series

To capture seasonal growth patterns, the growth trend for each individual at “n” days lag was assessed by calculating the slope of daily BAI_tree_ growth at a given time lag, from 2 to 100 days. For each time lag, we obtained the tree growth trend (in mm^2^ day^-1^). Then, for each temporal lag we assessed the average Spearman’s correlation between the tree growth series, to detect the time lag that maximized the coordination among the growth signal among trees (maximum average correlation) and minimized the information loss (maximum number of growth trend observations). Specifically, the optimum time lag was calculated following the Equation 7


(7) $$Opt_{i}=Cor_{i}\;*\;\frac{Nreg_{i}}{Nreg_{max}}$$

Where Opt_i_ is the score for the optimality function at a time lag “i”, Cor_i_ is the median Spearman’s correlation coefficient among time series at lag “i”, and Nreg_i_ and Nreg_max_ are the number of registers available at time lag “i” and the maximum number of registers, respectively.

**Figure 2:**
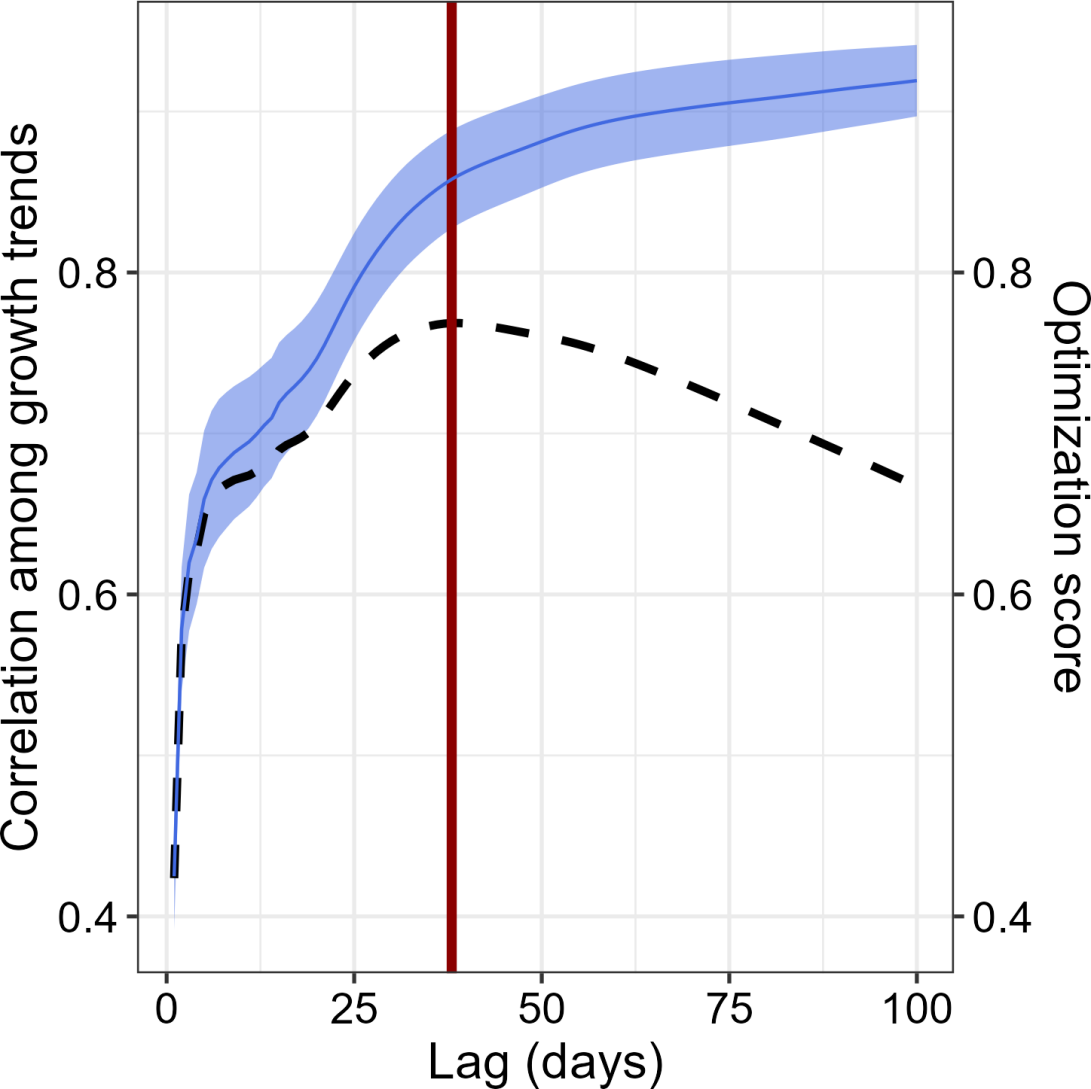
Correlation of the growth trends among the four *P. reticulata* trees in relation to time lag integrated, and optimization assessment. Correlation among the time-series signal increases with increasing number of days integrated, whereas the amount of information decreases, hence the shape of the optimization function (dashed black line). Accordingly, the optimal temporal resolution was determined to be at a lag of 38 days – vertical red line.

We identified the optimum time lag at 38 days (**Figure 2**). We then integrated growth trend and E_tree_ using the same time lag, and obtained the meteorological conditions within the given temporal lag accordingly (see **Table 1** for the relevant meteorological variables considered). Finally, to obtain its seasonal variations, we calculated the WUE_BAI_ for each tree at a 38-day time lag by dividing the cumulated BAI_tree_ by the cumulated E_tree_, during the same time lag. To reduce uncertainty related to tree size in both BAI_tree_ and WUE_BAI_, we standardized both variables at a tree level to correlate them to the environmental drivers.

**Table 1:** List of 38-days aggregated meteorological variables, their abbreviation and their units.

Meteorological variable	Abbreviation	Units
Average maximum temperature	MaxT	°C
Average minimum temperature	MinT	°C
Sum of degrees day	DD	°C
Average minimum relative humidity	Rh_min_	%
Average daily radiation	Q_day_	W m^-2^
Average daily photosynthetic active radiation	PARday	µmol m^-2^ s^-1^
Average wind speed	W_s_	m s^-1^
Number of hours with PAR > 250 µmol m^-2^ s^-1^	PAR > 250	unitless
Sum of potential evapotranspiration	CumPET	mm
Average potential evapotranspiration	MeanPET	mm day^-1^
Sum of precipitation	CumP	mm
Average vapor pressure deficit	MeanVPD	kPa

## 3 Results

### 3.1 Growth and WUE_BAI_ dynamics

**Figure 3:**
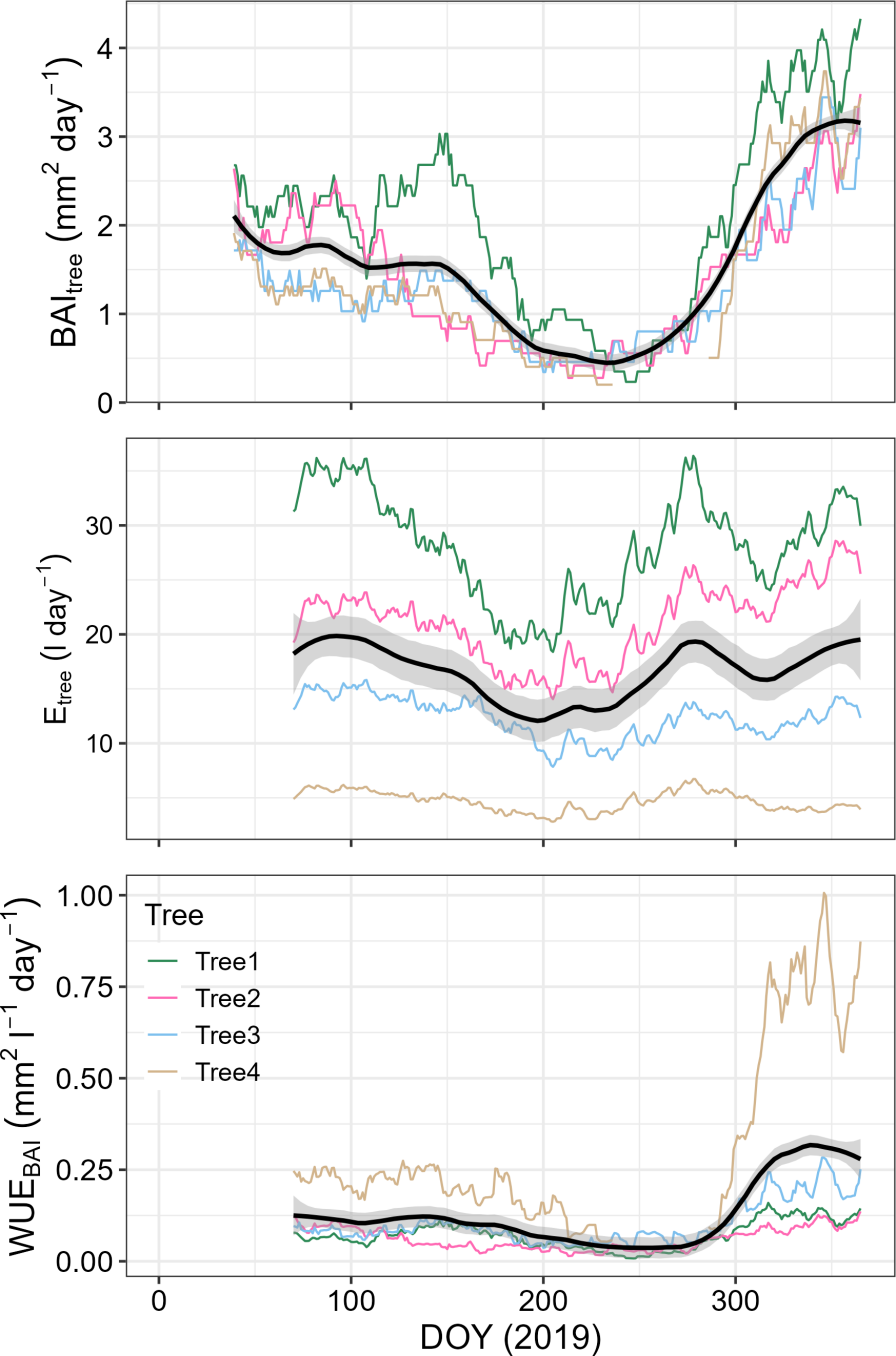
BAI_tree_, E_tree_ and WUE_BAI_ trends for *P. reticulata* during 2019. For the four *P. reticulata* studied trees, data is presented integrated in a time lag of 38 days. Noted are the trends for each individual, and black lines represent the average values plus a standard deviation.

**Figure 4:**
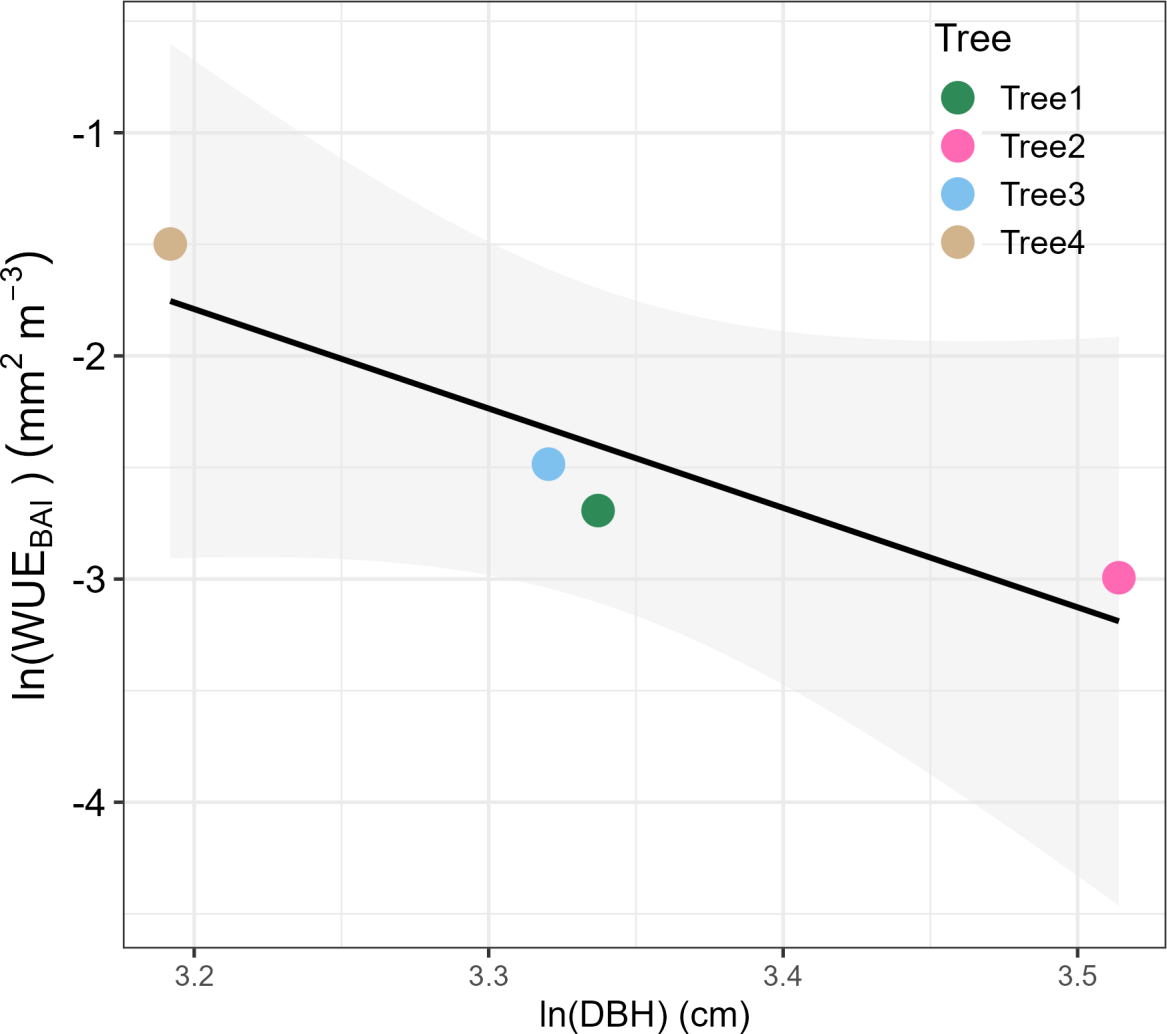
Relationship between the median water use efficiency in basal area increment (WUE_BAI_) with tree diameter at breast height (DBH) for the four *P. reticulata* trees studied, both transformed as Napierian logarithms to linearize the relationship.

By the end of 2019, *P. reticulata* trees had experienced an average basal area growth of 6.6 ± 1.8SE cm^2^, and transpired an average of 5.5 ± 3.4SE m^3^. Stem growth mostly occurred during August-May, when we also found the highest transpiration rates (**Figure 3**). Annual median WUE_BAI_ was related to tree size, with the larger trees being somewhat less efficient in transforming the water transpired into stem growth than the smaller ones (F=9.76, n = 4, p-value = 0.089, **Figure 4**).

**Figure 5:**
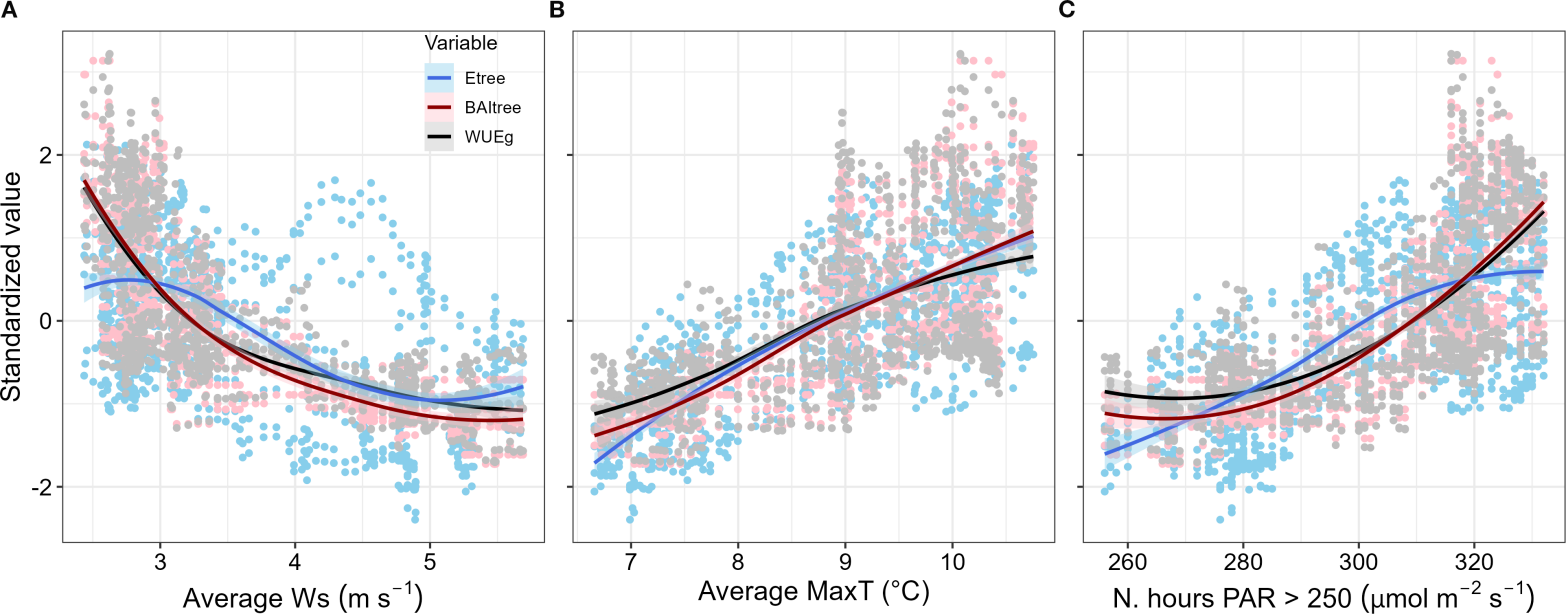
Correlations for E_tree_, BAI_tree_ and WUE_BAI_ with environmental variables. All variables were calculated with a 38 days lag, for the four *P. reticulata* trees monitored. **(A)** Average wind speed during the last 38 days, in m s^-1^; **(B)** average maximum temperature during the last 38 days, in °C; and **(C)** sum of the number of hours with PAR > 250 µmol m^-2^ s^-1^ during the last 38 days. Regression trend lines indicate the results of a “loess” spline fit ( ± 1SE) among the explanatory variables (MaxT, Ws and PAR > 250) and the standardized responses of tree transpiration (E_tree_), tree basal area growth (BAI_tree_), and water use efficiency in growth (WUE_BAI_).

**Figure 6:**
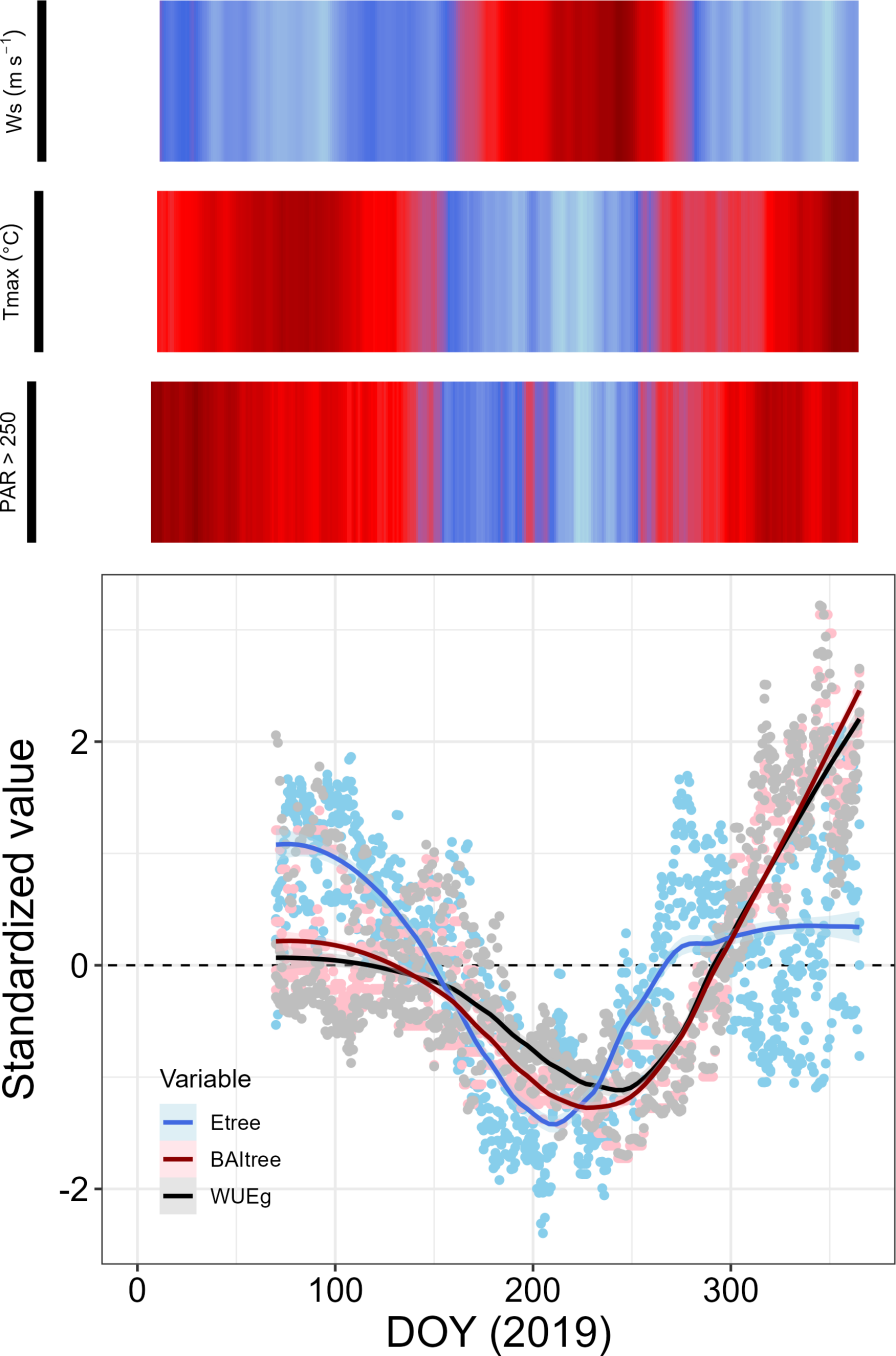
Standardized E_tree_, BAI_tree_ and WUE_BAI_ dynamics in *P. reticulata*, shown against the relevant environmental variables that explain their seasonal behavior. Values for each variable represent the tree-level standardized 38-days’ time lag trend, related to the environmental drivers: Wind speed (Ws), maximum temperature (Tmax), and number of hours with photosynthetic active radiation above 250 µmol m^-2^ s^-1^. All environmental drivers were also calculated at a 38-days’ time lag trend.

As with WUE_BAI_, E_tree_ and BAI_tree_ also exhibited strong seasonal trends. Moreover, WUE_BAI_ was more closely related to changes in BAI_tree_ (Spearman’s r = 0.971) than in E_tree_ (Spearman’s r = 0.179), (**Figure 5**). The seasonal trend found for E_tree_, BAI_tree_ and WUE_tree_ was determined mainly by the 38-previous days average wind speed, maximum temperature, and total number of hours with PAR greater than 250 µmol m^-2^ s^-1^ (**Figure 5**). We found that E_tree_, BAI_tree_ and WUE_tree_ decreased during periods with low temperatures and low irradiation, as well as high wind speed (**Figure 6**). Interestingly, low energy availability initially limited transpiration, whereas growth decline was delayed about ~ 20 days after transpiration decline (**Figure 6**). We also assessed the role of other environmental variables (i.e., relative humidity, precipitation and soil water content), though no apparent relation with E_tree_, BAI_tree_ or WUE_BAI_ appeared (see **Supplementary Figures S2**, **S3** in **Supplementary Material**).

As seen in **Figure 6**, we distinguished three different periods with contrasting phenological status within the year: (1) from DOY 32 to 150, with high E_tree_ values and medium BAI_tree_ and WUE_BAI_ values, (2) from DOY 151 to 240, with low values of E_tree_, BAI_tree_ and WUE_BAI_, and (3) from DOY 241 to the end of the year, when E_tree_ values started to increase first, followed by BAI_tree_ and WUE_BAI_. Finally, BAI_tree_ values exceeded E_tree_ values, probably due to an increase in WUE_BAI_ during this period, since E_tree_ values were similar to those found during the first period. The specific mean values for the three stages can be found in **Supplementary Table S2** in **Supplementary Material**.

### 3.2 Correlation between plant traits

**Figure 7:**
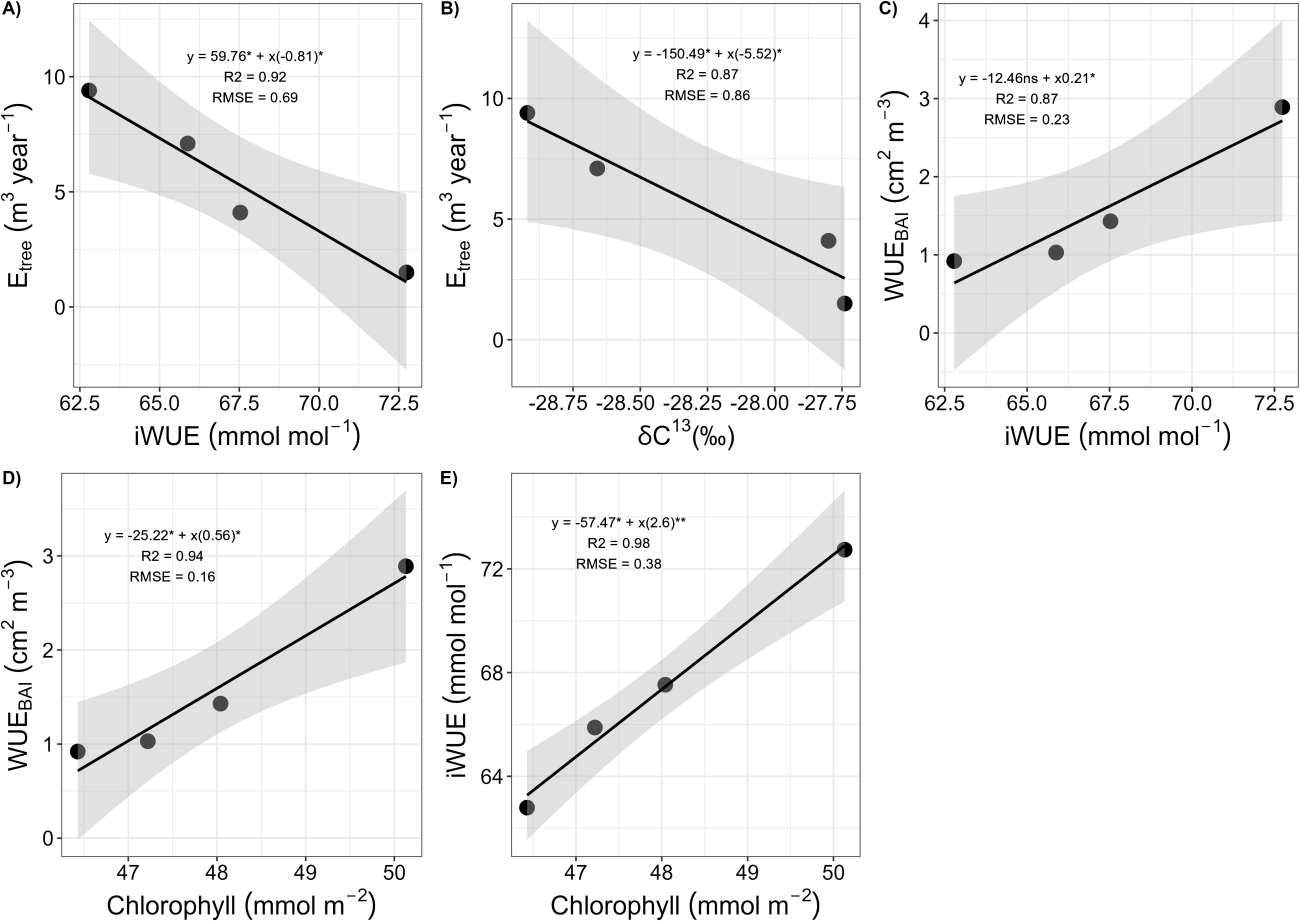
Correlations between E_tree_, WUE_BAI_ and iWUE with δ ^13^C_leaf_ and chlorophyll plant traits for the four *P. reticulata* trees monitored. **(A)** Relationship between the cumulated daily transpiration (E_tree_, m^3^ year^-1^) and intrinsic water use efficiency (iWUE, μmols mol^-1^); **(B)** Relationship between the cumulated daily transpiration (E_tree_, m^3^ year^-1^) and leaf δ^13^C (δ^13^C_leaf_, ‰); **(C)** Relationship between the water use efficiency in basal area increment (WUE_BAI_, cm^2^ m^3^) and intrinsic water use efficiency (iWUE, μmols mol^-1^); **(D)** Relationship between the water use efficiency in basal area increment (WUE_BAI_, cm^2^ m^3^) and chlorophyll (mmol m^-2^); and **(E)** Relationship between the intrinsic water use efficiency (iWUE, μmols mol^-1^) and chlorophyll (mmol m^-2^), for the four *P. reticulata* trees studied. For all figures, the regression line is in black, and the grey shadow represents SD and * represents significance of correlation at p-value < 0.05.

Measured *P. reticulata* life traits for both trees located at the interior and the exterior of the patch are shown on **Table 2**. For all trees combined, correlations between E_tree_ and iWUE, WUE_BAI_ and iWUE, iWUE and chlorophyll, E_tree_ and δ^13^C_leaf_ and WUE_BAI_ and chlorophyll are shown in **Figure 7**. E_tree_ showed a strong negative relationship with iWUE (b = -0.8, R^2^ = 0.92, p-value < 0.05), (**Figure 7A**), and with δ^13^C_leaf_ (b = -5.52, R^2^ = 0.86, p-value < 0.05) (**Figure 7B**). WUE_BAI_ showed a positive relationship with iWUE (b = 0.21, R^2^ = 0.87, p-value < 0.05) (**Figure 7C**) and chlorophyll (b = 0.56, R^2^ = 0.94, p-value < 0.05) (**Figure 7D**), and we also found a positive relationship between iWUE and chlorophyll (b = 2.6, R^2^ = 0.98, p-value < 0.01) (**Figure 7E**). Combined, those traits indicate a tight coordination between intrinsic water use efficiency and water use efficiency in growth, and both of them being dependent on leaf chlorophyll content.

**Table 2:** Description of *P. reticulata* tree, leaf and ecophysiological traits.

Trait	N	Edge trees	Interior trees
DBH (cm)	7	33.6 ± 8.8	35.7 ± 8.2
Basal area (m^2^)	7	0.1 ± 0.05	0.1 ± 0.05
Tree height (m)	7	8.3 ± 3.5	9.4 ± 1.8
WD (g cm^-3^)	7	0.54 ± 0.04	0.54 ± 0.04
Sapwood area (m^2^)	7	0.05 ± 0.03	0.06 ± 0.06
Leaf area (cm^2^)	140	3.64 ± 1.69	3.81 ± 0.42
Leaf thickness (mm)	140	0.55 ± 0.06	0.52 ± 0.13
SLA (mm^2^ mg^-1^)	140	4.34 ± 0.4	4.54 ± 0.5
Chlorophyll (mmol m^-2^)	140	0.57 ± 0.04	0.57 ± 0.04
N_leaf_ (%)	6	1.56 ± 0.20	1.52 ± 0.08
C_leaf_ (%)	6	49.4 ± 2.7	50.1 ± 1
δ^13^C_leaf_ (‰)	6	-28.3 ± 0.5	-28 ± 0.6
Δ^13^C_leaf_ (‰)	6	0.21 ± 0.01	0.20 ± 0.01
E_tree_ (m^3^ year^-1^)	4	5.5 ± 3.4	–
BAI (cm^2^ year^-1^)	4	6.6 ± 1.8	–
WUE_BAI_ (cm^2^ m^3^)	4	1.2 ± 0.1	–
iWUE (μmols mol^-1^)	6	60.7 ± 5.3	66.6 ± 6.3
WP_PD_ (MPa)	7	0.15 ± 0.02	0.20 ± 0.01
WP_MD_ (MPa)	7	1.00 ± 0.39	1.20 ± 0.41
N_SOIL_ (%)	5	0.91 ± 0.20	1.08 ± 0.25
C_SOIL_ (%)	5	15.2 ± 1.7	16.1 ± 1.9
C/N_SOIL_	5	19.9 ± 5.1	17.6 ± 4.8

## 4 Discussion

There is an ongoing debate about whether tree growth cessation is driven by C-source limitations (e.g., reduced in photosynthetic activity under stress) or by C-sink limitations (e.g., inhibition of cambial cell differentiation under limiting conditions, such as low water potential or temperature) (Körner, 2015; Trugman and Anderegg, 2025). During year 2019, *Polylepis reticulata* exhibited a highly temperature-dependent stem growth behavior, indicative of sink-related growth limitations (Cabon et al., 2022). This was even more evident in its water use efficiency in growth (WUE_BAI_) dynamics, which were much more dependent on stem growth than on transpiration changes along the year. This underscores that under the prevailing environmental conditions *P. reticulata*’s water management is not driven by strategies to conserve water due to water shortage, but rather it is driven by reductions in stem growth potential due to low temperatures. Thus, water shortage has probably not exerted a strong selective pressure on *P. reticulata*’s physiology. This was further confirmed by its low responsiveness to declining soil water content (**Supplementary Figure S3** in **Supplementary Material**), and its low intrinsic water use efficiency (iWUE) determined from its leaf isotopic δ^13^C_leaf_ fractioning, with average values of ca. 60 µmol CO_2_ mol^-1^ H_2_O that match the average values reported for tropical and subtropical moist broadleaf forests (Mathias and Thomas, 2021).

### 4.1 Growth seasonality at the Andean tree line

Our results show that *P. reticulata* presents an intra-annual stem growth pattern determined by the seasonality of the environmental drivers, especially temperature and irradiance. We found that growth was enhanced during periods with higher temperature and PAR, whereas no relationship was found between growth and precipitation, relative humidity or soil water content, suggesting that growth is mainly determined by energy availability rather than water availability. This agrees with previously reported Ecuadorian páramo energy-limited conditions (Carabajo-Hidalgo et al., 2023).

Trees in Ecuadorian regions usually do not present visible tree-rings due to low seasonality (Bräuning et al., 2009), but Alvites et al. (2019) were able to distinguish some growth rings in *P. reticulata* samples. Nevertheless, they did not detect clear relationships between tree-ring width and climate, despite some samples showing a positive relationship between ring width and monthly average temperature. The strong growth seasonality signal found in our study hints the possibility of a more subtle signal present in the wood anatomy, in line with previous findings (Makinen et al., 2003; Deslauriers et al., 2017; García-Cervigón et al., 2020), that could potentially allow to date tree annual growth in *P. reticulata* through the analysis of xylem cells anatomical variation, paving the way for future studies on climate variations at the top of the Andean tree line. Nevertheless, due to the technical limitations of measuring a tree species that grows at ~4000 m a.s.l. in the Andes, our field campaigns consist of only one year of continuous measurements. Additional campaigns with a more extended measurement period would likely shed more light on *P. reticulata*’s growth and transpiration phenology, and bolster the robustness of our observations.

### 4.2 Environmental constraints on tree growth

The observed temperature dependence of growth, combined with previously reported high photosynthetic potential of *P. reticulata* (Carabajo-Hidalgo et al., under review), suggest that growth is not limited by carbon assimilation (source limitation), but by tissue growth itself (sink limitation). High-altitude plants adapted to cold climates are able to perform photosynthesis at low temperatures (García-Plazaola et al., 2015), but the carbon gained is mostly accumulated as non-structural carbohydrates, to be then mobilized to the active tissues when temperature is not as limiting. This means that, although photosynthesis may occur at low temperatures, meristem cell duplication ceases (Körner, 2015), stopping growth below 6°C. Also, the growth limitation at higher wind speeds conditions can be related to the plant-to-environment heat exchange. Higher wind speeds would break the boundary layer surrounding plant tissues more easily, resulting in lower effective temperatures that can limit both photosynthesis and cambium cell division (Zhang et al., 2021). On the contrary, during periods with comparatively higher temperatures and low wind speed, the aforementioned constraints disappear and *P. reticulata*’s growth increases. It is worth mentioning that here we addressed the growth and water use efficiency patterns in a single year, in which temperature, wind speed and irradiation changes were highly correlated during the coldest season. Further observations on the effects of these drivers during years with less homogeneous changes in the behavior of the three environmental drivers would provide additional insight on their individual importance on *P. reticulata*’s growth patterns.

### 4.3 Intra-annual water use efficiency driven by stem growth patterns

We found that smaller trees were the most efficient in transforming transpired water to stem growth, while larger trees were the least efficient, perhaps due to their higher canopy exposure to wind. Wind can break the boundary leaf layer and alter transpiration rates, photosynthesis and growth by triggering changes in stomatal conductance and leaf temperature (Burgess et al., 2016). In addition, the higher WUE_BAI_ rates began on DOY 240 (from August onwards), when more favorable conditions, such as higher temperature, higher PAR and lower wind speed, resulted in growth exceeding transpiration and, consequently, an increase in WUE_BAI_. WUE_BAI_ was strongly related to growth dynamics rather than transpiration (**Figure 5**), suggesting that sink limitation due to low temperatures is the key limiting factor for *P. reticulata*’s growth.

### 4.4 Morphological adaptations to environmental conditions

*Polylepis reticulata’s* life traits show typical high-altitude characteristics (Liu et al., 2020), meeting the general pattern of decreasing tree height and aboveground structures with altitude due to lower temperatures and atmospheric pressure and higher irradiance (Macek et al., 2009). In a recent publication Carabajo-Hidalgo et al. (2023) found transpiration rate differences between edge and interior trees for the same stand. In our analysis, we measured only trees growing at the edge of the patch. In these, differences in exposition due to tree size (e.g. **Figure 4**) may have influenced our measurements. This likely indicates that *P. reticulata*’s size is a strong driver of its ecophysiological response to environmental drivers, with dense patches likely acting as a buffer for the inner trees, least exposed to local climate changes. In other *Polylepis* forests differences in plant traits were found following an exposure gradient, suggesting that edge trees adjusted their functional traits to adapt to worse weather conditions, such as high maximum solar radiation under clear-sky conditions, wind-induced cooling, and low night temperatures (Ramos et al., 2013).

When we compare *P. reticulata* traits with those of other *Polylepis* species, results are highly variable depending on the prevailing environmental conditions. *P. reticulata* trees growing in the Ecuadorian páramo ecosystem are taller than *P. pepei* trees from the Bolivian Andes, which is a colder environment (Hertel and Wesche, 2008), and they are also taller than other *Polylepis* species developing in puna ecosystem, which is a much drier environment (Macek et al., 2009). This suggests that both temperature and water have an important role in shaping tree height. Low temperature and high precipitation increase leaf thickness and SLA, respectively (Toivonen et al., 2014), which explains the higher values found for these *P. reticulata* traits when compared with *Polylepis* from puna ecosystem. Finally, there is a positive relation between δ^13^C and iWUE (Bauters et al., 2020), and higher δ^13^C values point to greater drought stress. Values for *P. reticulata* were lower than those found for *Polylepis* species growing in drier ecosystems (Macek et al., 2009), which suggests that *P. reticulata* was not exposed to drought stress, as expected for páramo ecosystem.

Regarding iWUE and WUE_BAI_ we found them to be highly correlated. Also, both of them were positively related with chlorophyll content. This can be explained by an increase in the photosynthetic rate at the tree line due to chlorophyll increment, which in turn results in higher photosynthesis potential, resulting in increased water use efficiency, both at the leaf and at the tree level (Pu and Lyu, 2023). Lastly, some studies related higher leaf δ^13^C with higher drought stress (Bauters et al., 2020; Ren et al., 2024), which usually implies progressive stomatal closure and decreasing in E_tree_. In accordance, we found a negative relationship between E_tree_ and δ^13^C_leaf_, which is the expected in C_3_ plant species (Sorgini et al., 2021).

### 4.5 Will global warming enhance *P. reticulata*’s growth?

IPCC projections for the Ecuadorian Andes project an increase in atmospheric CO_2_ and temperature, as well as changes in precipitation distribution, for the 21^st^ Century (Urrutia and Vuille, 2009; Mora et al., 2014). Such conditions could potentially enhance *P. reticulata*´s growth, as we found that the main limitation for its growth is the low energy availability. However, despite higher energy availability could boost *P. reticulata*´s growth in the short term, it is still not clear whether an increase in water stress will become a more important limiting factor for *P. reticulata* in the future. Together, projected increases in evaporative demand water shortage could endanger *P. reticulata* growth, as this species is likely not adapted to deal with water scarcity (Novick et al., 2024), potentially offsetting the advantages of higher temperatures and radiation incidence during the cold season.

## 5 Conclusions

Stem growth of *P. reticulata* trees growing at the Ecuadorian páramo presents a marked intra-annual seasonality, due to growth limitations resulting from low temperatures and low radiation during the cold season. This translates into large variations in seasonal water use efficiency, mostly driven by reductions in stem growth. This marked temperature-driven seasonality clearly indicates a carbon-sink limitation to growth for *P. reticulata*, highlighting the harsh conditions that trees growing at such high altitudes face, especially with low temperatures limiting cambium cell differentiation. Accordingly, *P. reticulata*’s life traits apparently do not prioritize an efficient water use. This implies that the growth of such species should be extremely sensitive to increases in temperatures due to global warming, with key uncertainties regarding its response if accompanied by declining soil water content and reduced nutrient availability.

## Data Availability

The raw data supporting the conclusions of this article will be made available by the authors, without undue reservation.
