# Supplementary material for: Cold and low irradiation shape Polylepis reticulata’s seasonal growth and water use dynamics at the Ecuadorian Andean tree line
Source: Front Plant Sci. 2025 Oct 13;16:1675655. doi: 10.3389/fpls.2025.1675655 (PMC12555009; doi:10.3389/fpls.2025.1675655)
Supplement: Supplementary file 1 [file DataSheet1.pdf]

Cold and low irradiation shape *Polylepis reticulata*'s seasonal growth and water use dynamics at the Ecuadorian Andean tree line

Aldemar Carabajo-Hidalgo[1,2,3;*], Daniel Nadal-Sala[2,3;*], Byron Poma[1], Heidi Asbjornsen[4], Patricio Crespo[1] and Santiago Sabaté[2,3]

[1]Departamento de Recursos Hídricos y Ciencias Ambientales, Facultad de Ingeniería, Universidad de Cuenca, Campus Balzay, Cuenca, 010107, Ecuador; [2]Departamento de Biología Evolutiva, Ecología y Ciencias Ambientales, Universitat de Barcelona, Av. Diagonal, 643, 08028, Barcelona, Spain; [3]CREAF (Centre de Recerca i Aplicacions Forestals), Campus de Bellaterra (UAB) Edifici C., Cerdanyola del Vallès, 08193, Spain; [4]Department of Natural Resources and the Environment and Earth Systems Research Center, University of New Hampshire, 114 James Hall, Durham, NH 03824, USA.

Supplementary Material

1 Supplementary Figures and Tables

Supplementary Table S1. Initial DBH for the four studied trees.

Tree	Initial DBH (cm)
Tree 1	33.5
Tree 2	28.1
Tree 3	27.8
Tree 4	24.2

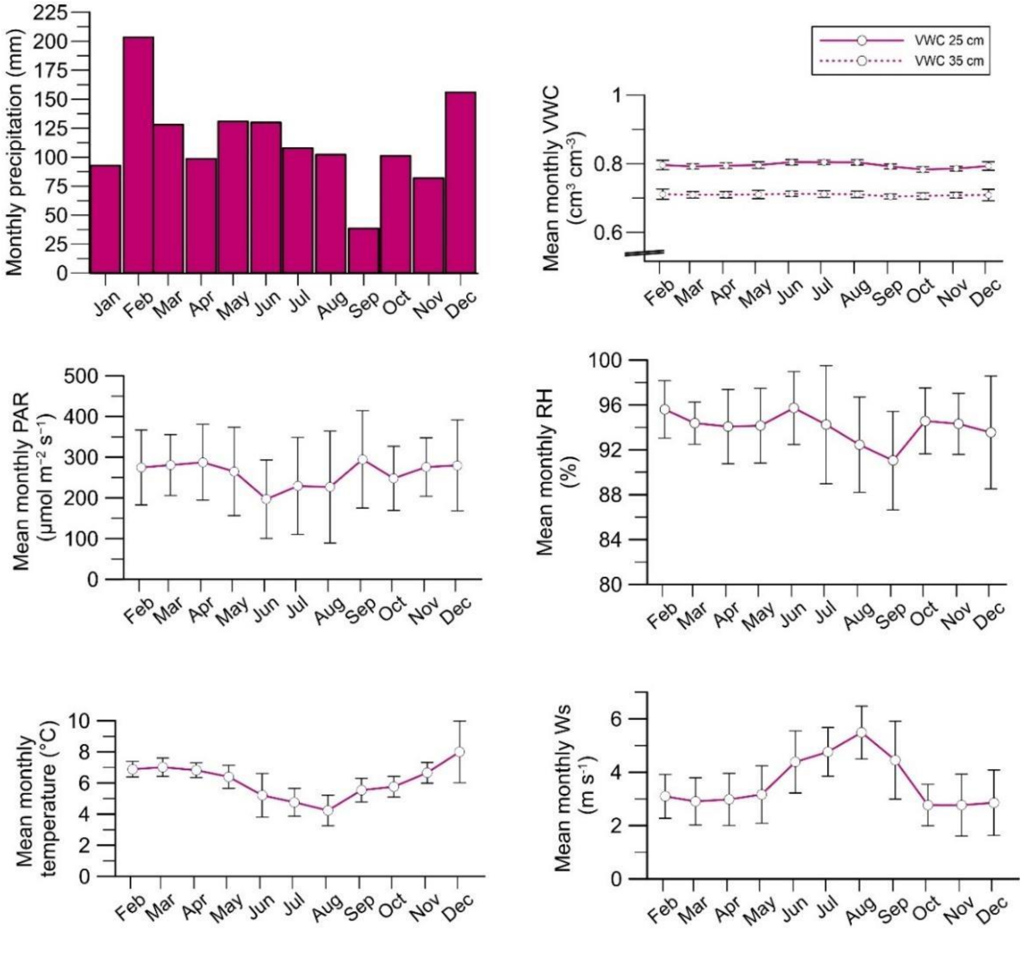

Supplementary Figure S1. Mean monthly values for precipitation, VWC, PAR, RH, temperature and Ws for the study period (2019).

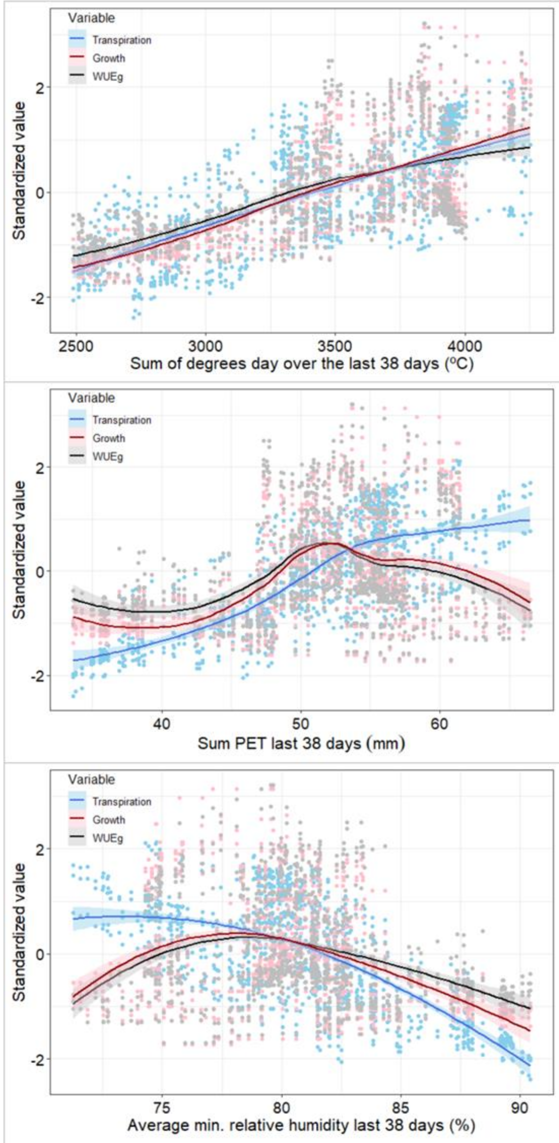

Supplementary Figure S2. Correlations for E_{tree}, BAI_{tree} and WUE_{BAI} with environmental variables with a 38 days temporal lag, and for the four *P. reticulata* trees studied. Above) sum of degrees per day, in °C; middle) sum of precipitation, in mm; and below) average minimum relative humidity, in %. Regression trendlines indicate the results of a "loess" spline fit (±1SE) among the explanatory variables (Temperature, Precipitation and Relative humidity) and the standardized responses of tree transpiration (E_{tree}), tree basal area growth (BAI_{tree}), and water use efficiency in growth (WUE_{BAI}).

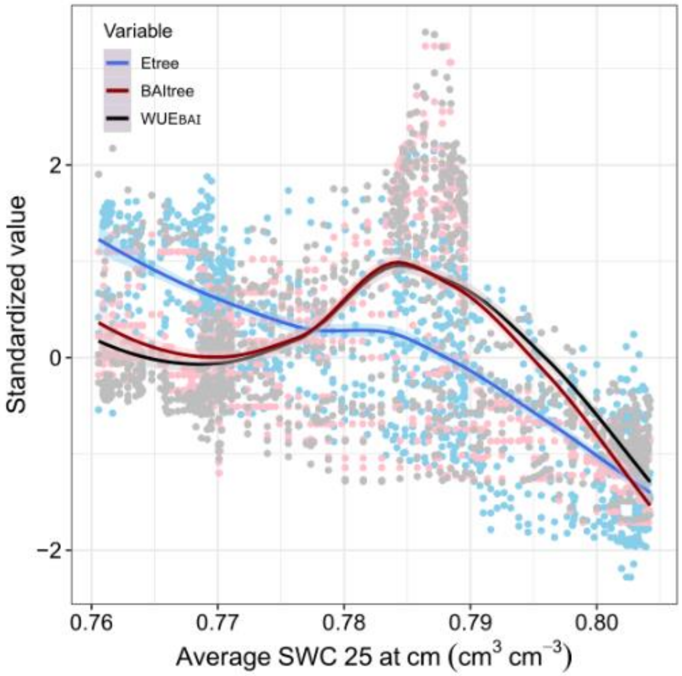

Supplementary Figure S3. Standardized responses of 38-days integrated transpiration (E_{tree}), stem growth (BAI_{tree}) and water use efficiency in growth (WUE_{BAI}) to average soil water content (SWC) at 25 cm depth, for four *P. reticulata* trees growing at páramo, Ecuador, during the year 2019. Dots indicate the aggregated daily measurements, and trendlines indicate a "loess" fit to show the trend of the relationship.

Supplementary Table S2. Mean and standard deviation values for BAI_{tree} per day, E_{tree} per day and WUE_{BAI} during the entire 2019 year, and also for three different periods within the year, consisting to three different phenological stages.

Periods	BAI_{tree} (mm^2 day^{-1})	E_{tree} (dm^3 day^{-1})	WUE_{BAI} (cm^2 m^{-3})	n
	Mean ± SD	Mean ± SD	Mean ± SD	
**2019_{year}**	1.97 ± 0.05	16.47 ± 0.10	1.20 ± 0.05	4
**$32\text{-}150_{DOY}$**	1.68 ± 0.05	17.80 ± 0.11	0.94 ± 0.11	4
**$151\text{-}240_{DOY}$**	1.67 ± 0.12	12.36 ± 0.08	1.35 ± 0.13	4
**$241\text{-}365_{DOY}$**	2.58 ± 0.06	18.55 ± 0.12	1.39 ± 0.16	4
